# H. C. Peebles

**Published:** 1871-07

**Authors:** 


### OBITUARY NOTICES.

H. C. Peebles, D.D.S., was born in Virginia in 1812, and died in St. Louis Missouri, in February 1871. Dr. Peebles commenced the study of dentistry in Ohio, from whence he removed to Missouri, and practiced for a number of years in the town of Lexington, where he enjoyed a high reputation. Removing to St. Louis, he occupied a prominent position among the dental practitioners of that city until his heath.

In 1850, he received the degree of Doctor of Dental Surgery, from the Baltimore College of Dental Surgery. In an obituary notice, Dr. Judd speaks as follows of his former colleague, which all who were acquainted with the late Dr Peebles will endorse.

"Dr Peebles was a man of great energy of character, and having early imbibed correct views in regard to dental education, he entered into the discussion of the educational questions of the day with spirit, and ceased only with his life to exert all his influence for bringing about a higher grade of literary and scientific attainments for the members of the dental profession.

When the Missouri Dental College was organized, he entered upon the duties of a teacher in that Institution, which he filled with credit to himself and to the college, until his failing health admonished of the necessity of resigning a position, the onerous duties of which he was no longer able to perform. The organization of the Missouri State Dental Association was due to his almost unaided efforts, and for many years he was an active member of the St. Louis Dental Society, and of the Western Dental Association, and also of the National Dental Association.

No member of the profession could have been taken from its ranks in Missouri, whose loss would have been more severely felt. He leaves a family and many friends to deplore his loss; and an example whose benign influence shall be felt through many a coming year." "Blessed are the dead who die in the Lord for their works do follow them."

